# Baicalin promotes the viability of Schwann cells *in vitro* by regulating neurotrophic factors

**DOI:** 10.3892/etm.2021.11056

**Published:** 2021-12-13

**Authors:** Wenpu Zuo, Huayu Wu, Kun Zhang, Peizhen Lv, Fuben Xu, Weizhe Jiang, Li Zheng, Jinmin Zhao

Exp Ther Med 14:507–514, 2017; DOI: 10.3892/etm.2017.4524

After the publication of the above article, the authors have realized that Figs. 3, 5 and 7 in their paper were published with errors; regarding Fig. 3, the data panel for the ‘2 days, 5 µM baicalin’ experiment was selected incorrectly, whereas with Figs. 5 and 7, an incorrect data panel was selected for the ‘Control, 4 days’ data. These errors arose inadvertently as a consequence of the authors’ misfiling of their data.

The revised versions of Figs. 3, 5 and 7, featuring the corrected data panels for the above-mentioned experiments, are shown opposite. Note that the revised data shown for these Figures do not affect the overall conclusions reported in the paper. The authors apologize to the Editor of *Experimental and Therapeutic Medicine* and to the readership for any inconvenience caused.

## Figures and Tables

**Figure 3:**
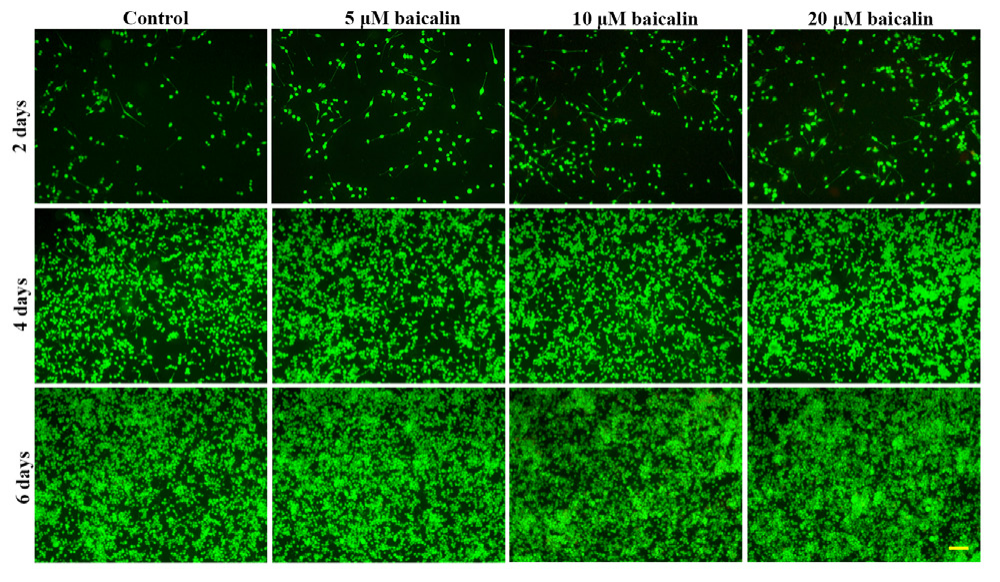
Cell viability was measured by fluorescein diacetate staining under a light microscope. RSC96 Schwann cells were incubated with 0 (control), 5, 10 or 20 µM baicalin for 2, 4 and 6 days (magnification, x100; scale bar, 200 µm).

**Figure 5:**
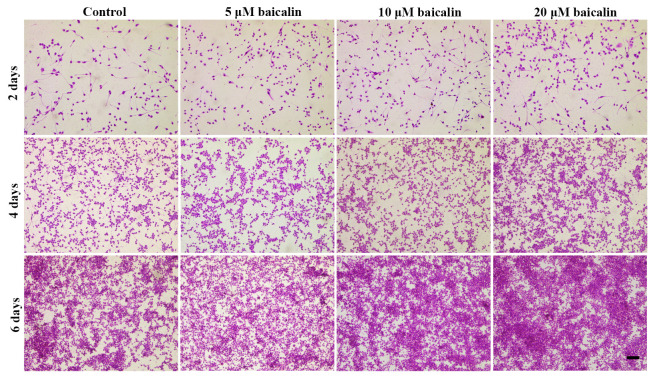
Hematoxylin-eosin staining images showing the morphology of RSC96 SCs cultured *in vitro* with 0 (control), 5, 10 or 20 µM baicalin for 2, 4 and 6 days (magnification x100; scale bar, 200 µm).

**Figure 7:**
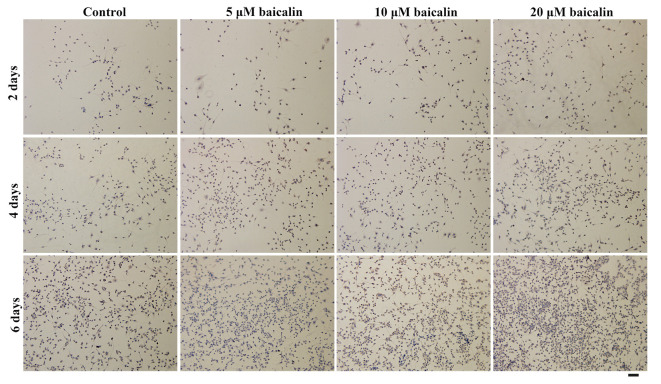
Immunohistochemical staining images revealed the presence of S100β. RSC96 Schwann cells were cultured *in vitro* with 0 (control), 5, 10 or 20 µM baicalin for 2, 4 and 6 days (magnification x100; scale bar, 200 µm).

